# Reappraisal of *Immotthia* in Dictyosporiaceae, Pleosporales: Introducing *Immotthia bambusae* sp. nov. and *Pseudocyclothyriella clematidis* comb. et gen. nov. Based on Morphology and Phylogeny

**DOI:** 10.3389/fmicb.2021.656235

**Published:** 2021-05-07

**Authors:** Hong-Bo Jiang, Rajesh Jeewon, Samantha C. Karunarathna, Chayanard Phukhamsakda, Mingkwan Doilom, Pattana Kakumyan, Nakarin Suwannarach, Rungtiwa Phookamsak, Saisamorn Lumyong

**Affiliations:** ^1^CAS Key Laboratory for Plant Diversity and Biogeography of East Asia, Kunming Institute of Botany, Chinese Academy of Sciences, Kunming, China; ^2^Center of Excellence in Fungal Research, Mae Fah Luang University, Chiang Rai, Thailand; ^3^School of Science, Mae Fah Luang University, Chiang Rai, Thailand; ^4^Department of Health Sciences, Faculty of Medicine and Health Sciences, University of Mauritius, Réduit, Mauritius; ^5^Research Center of Microbial Diversity and Sustainable Utilization, Faculty of Sciences, Chiang Mai University, Chiang Mai, Thailand; ^6^CIFOR-ICRAF China Program, World Agroforestry (ICRAF), Kunming, China; ^7^Honghe Center for Mountain Futures, Kunming Institute of Botany, Chinese Academy of Sciences, Honghe, China; ^8^Centre for Mountain Futures (CMF), Kunming Institute of Botany, Kunming, China; ^9^Engineering Research Center of Chinese Ministry of Education for Edible and Medicinal Fungi, Jilin Agricultural University, Changchun, China; ^10^Innovative Institute of Plant Health, Zhongkai University of Agriculture and Engineering, Guangzhou, China; ^11^Department of Biology, Faculty of Science, Chiang Mai University, Chiang Mai, Thailand; ^12^Academy of Science, The Royal Society of Thailand, Bangkok, Thailand

**Keywords:** bambusicolous fungi, Dothideomycetes, *Pseudocoleophoma*, taxonomy, two new taxa

## Abstract

*Immotthia* is a poorly known genus, and currently, no DNA sequence data are available to ascertain its proper phylogenetic placement and evolutionary relationships with other bitunicate fungi. To date, there are only two species accepted in the genus. During our ongoing research study of bambusicolous fungi in southwest China and Thailand, a fungus associated with stromata of *Hypoxylon* sp. was found on dead bamboo culms in Loei Province, Thailand. Preliminary morphological identification revealed that the fungal collection belongs to *Immotthia*. A novel species, *Immotthia bambusae*, is introduced herein based on a comparison of morphological characteristics with the type specimen of *I. hypoxylon* (≡ *Amphisphaeria hypoxylon* Ellis and Everh.), a synonym of *I. atrograna* (Cooke and Ellis) M. E. Barr. Phylogenetic analyses of a concatenated ITS, LSU, SSU, and TEF1-α DNA sequence matrix showed that *Immotthia* belongs to Dictyosporiaceae, Pleosporales. Despite *I. bambusae* strains constituting a supported subclade, they are nested with the genus *Pseudocoleophoma*. *Pseudocoleophoma clematidis* is morphologically different from all other *Pseudocoleophoma* species, while its conidial characteristics are similar to *Cyclothyriella*. Multigene phylogenetic analyses showed that *P. clematidis* formed a clade basal to *Immotthia*, separated from *Pseudocoleophoma* with strong statistical support. Therefore, we introduce a monotypic genus, *Pseudocyclothyriella* Phukhams. and Phookamsak, gen. nov. to accommodate the single species, *Pseudocyclothyriella clematidis* (Phukhams. and K. D. Hyde) Phukhams. and Phookamsak, comb. nov. Detailed descriptions, color micrographs, and phylogenetic trees to show the placement of the new taxa are provided. In addition, an updated taxonomic treatment of the genera *Immotthia* and *Pseudocyclothyriella* is also provided based on the study of the type materials and phylogeny generated from DNA sequence data.

## Introduction

*Immotthia* was introduced by Barr (1987a) with *I. hypoxylon* (Ellis and Everh.) M. E. Barr (≡ *Amphisphaeria hypoxylon* Ellis and Everh.) as the type species. Through examinations of the type material of *I. hypoxylon* and Australian collections of *I. atrograna* (Cooke and Ellis) M. E. Barr (≡ *Sphaeria atrograna* Cooke and Ellis), Jaklitsch et al. (2002) concluded that these two taxa are conspecific. To date, two species are accepted in this genus, viz. *I. atrograna* and *I. atroseptata* (Piroz.) M. E. Barr (Species Fungorum, 2020) based on morphology, but no DNA sequence data are available to confirm their phylogenetic placement (Hyde et al., 2017; Doilom et al., 2018).

*Immotthia* is characterized by small- to medium-sized, globose to subglobose ascomata, forming on blackened hypostroma, bitunicate, fissitunicate, cylindrical asci, and brown to reddish brown, ellipsoidal to fusiform, 1-septate, smooth or slightly verrucose ascospores (Hyde et al., 2017; Hongsanan et al., 2020). The asexual morph of *Immotthia* has been reported as coelomycetous, identified as *Coniothyrium parasitans* (Berk. and Ravenel) Tassi which formed enteroblastic, phialidic, doliiform to ampulliform, or cylindrical, smooth, hyaline conidiogenous cells bearing brown, ellipsoidal, smooth, and aseptate conidia (Hyde et al., 2017; Hongsanan et al., 2020). However, the link between *Immotthia* and *C. parasitans* has not yet been proven based on DNA sequence analyses. *Immotthia* has been reported as hyperparasites on stromata of *Annulohypoxylon*, *Hypoxylon*, and *Pestalopezia*, or forms compressed ascostromata on decorticated wood (Cooke and Ellis, 1879; Ellis and Everhart, 1886; Pirozynski, 1973; Jaklitsch et al., 2002; Akulov and Hayova, 2016; Hyde et al., 2017; Hongsanan et al., 2020).

*Immotthia* was assigned to Dacampiaceae (Pleosporales, Dothideomycetes) by Barr (1987a, b) and this taxonomic treatment was followed by Akulov and Hayova (2016). Barr (2002) transferred non-lichenicolous genera from the Dacampiaceae to Teichosporaceae (Pleosporales, Dothideomycetes), where *Immotthia* was also included along and transferred to Teichosporaceae. Recently, *Immotthia* was tentatively placed in Roussoellaceae (Pleosporales, Dothideomycetes) based on similar morphological features of the asci, ascospores, and coelomycetous asexual morph which largely resemble taxa in Roussoellaceae (Hyde et al., 2017; Doilom et al., 2018; Hongsanan et al., 2020).

A well-studied genus *Pseudocoleophoma* Kaz. Tanaka and K. Hiray. was introduced by Tanaka et al. (2015) to accommodate two novel species having asexual morphology similar to *Coleophoma* from Japan. The genus is characterized by scattered to clustered, immersed to erumpent, globose to subglobose ascomata, with ostiolar neck, thin-walled peridium, composed of brown to dark brown, polygonal to rectangular cells, cylindrical to clavate, fissitunicate asci, with short-pedicellate, and hyaline, fusiform, 1-septate ascospores, with a conspicuous sheath (Tanaka et al., 2015; Jayasiri et al., 2019). *Pseudocoleophoma* has coelomycetous, coleophoma-like asexual morphs forming pycnidial, subglobose conidiomata, phialidic, doliiform to lageniform conidiogenous cells, and cylindrical or oblong, hyaline, aseptate, smooth-walled conidia (Tanaka et al., 2015). The genus belongs to Dictyosporiaceae based on phylogenetic evidence (Tanaka et al., 2015).

Two holomorphic species, *Pseudocoleophoma calamagrostidis* Kaz. Tanaka and K. Hiray. and *P. polygonicola* Kaz. Tanaka and K. Hiray., were initially accommodated in this genus (Tanaka et al., 2015). Later, six other species were accommodated based on morphological and phylogenetic support, viz. *P. bauhiniae* Jayasiri, E. B. G. Jones and K. D. Hyde (on *Bauhinia* sp., Thailand); *P. clematidis* Phukhams. and K. D. Hyde (on *Clematis vitalba*, Italy); *P. flavescens* (Gruyter, Noordel. and Boerema) W. J. Li and K. D. Hyde (from soil, rhizosphere of *Solanum tuberosum*, Netherlands); *P. rusci* W. J. Li, Camporesi and K. D. Hyde (on *Ruscus aculeatus*, Italy); *P. typhicola* Kamolhan, Banmai, Boonmee, E. B. G. Jones and K. D. Hyde (on decaying submerged *Typha latifolia*, Great Britain); and *P. zingiberacearum* Tennakoon, D. J. Bhat, C. H. Kuo and K. D. Hyde (on *Hedychium coronarium*, Taiwan) (Tanaka et al., 2015; Hyde et al., 2016; Jayasiri et al., 2019; Tennakoon et al., 2019; Li et al., 2020; Phukhamsakda et al., 2020).

Most of the *Pseudocoleophoma* species have been represented by their asexual morphs (Hyde et al., 2016; Tennakoon et al., 2019; Li et al., 2020; Phukhamsakda et al., 2020). Only three species have been reported for both sexual and asexual morphs, viz. *P. bauhiniae*, *P. calamagrostidis*, and *P. polygonicola* (Tanaka et al., 2015; Jayasiri et al., 2019). Currently, species of *Pseudocoleophoma* are only known from Europe (Great Britain, Italy, and Netherlands) and Asia (Taiwan and Thailand), and they were found as saprobes on various hosts and substrates from both terrestrial and freshwater habitats (Tanaka et al., 2015; Hyde et al., 2016; Jayasiri et al., 2019; Tennakoon et al., 2019; Li et al., 2020; Phukhamsakda et al., 2020).

In the present study, a fresh collection of *Immotthia* is examined and compared with other *Immotthia* species based on morphological characteristics. The new collection is described as a novel species in *Immotthia* and illustrated. Through DNA sequencing of the fresh material, we also resolved the phylogenetic placement of *Immotthia* for the first time, based on maximum likelihood and Bayesian inference analyses. In addition, the novel genus *Pseudocyclothyriella* is also introduced to accommodate *Pseudocyclothyriella clematidis* comb. nov. (≡ *Pseudocoleophoma clematidis*) based on morphological distinctiveness and multigene phylogenetic analyses.

## Materials and Methods

### Sample Collection, Specimen Examination, and Preservation

Dead bamboo culms were collected from Loei Province, Thailand, in 2020. The specimens were kept in a paper bag and returned to the laboratory for observation and examination. Fungal fruiting bodies on the host substrate were observed with a Motic SMZ 140 series dissecting stereoscope, and a centrum was mounted in sterilized distilled water for morphological examination and captured using a Nikon ECLIPSE Ni compound microscope connected with a Canon EOS 600D digital camera. Tarosoft (R) Image Frame Work version 0.9.7 was used to measure the size of ascomata, peridium, pseudoparaphyses, asci, and ascospores. In addition, holotypic specimens of *Immotthia atroseptata* [United States, North Carolina, behind N. C. Department of Agriculture, Nursery Inspection Station, 1 mile west of Linville, Avery Co., on apothecia of *Pestalopezia rhododendri* on the leaves of *Rhododendron maximum* L. (Ericaceae), March 21, 1972, Neli Lapp DAOM 139001], *I. hypoxylon* (United States, Louisiana, Plaquemines Parish, on decaying wood, December 30, 1885, A. B. Langlois 138, NY0083004), and *Pseudocoleophoma clematidis* (Italy, Arezzo Province, Badia Tega—Ortignano Raggiolo, on dead aerial branch of *Clematis vitalba*, March 9, 2013, E. Camporesi, IT 1110, MFLU 16-0280) were also re-examined and illustrated. Adobe Photoshop CS6 software (Adobe Systems Inc., United States) was used to edit and provide the photographic plates based on captured pictures of the fungal structures. Good practices for morphological examinations as outlined by Senanayake et al. (2020b) were followed for the morphological study, while phylogenetic methods as outlined by Dissanayake et al. (2020) were followed for phylogenetic analyses. The holotype is deposited in the herbarium of Cryptogams Kunming Institute of Botany Academia Sinica (KUN-HKAS), Yunnan, China. The isotype was stored in Herbarium Mycologicum Academiae Sinicae (HMAS), Beijing, China. The Facesoffungi and Index Fungorum numbers are registered for the newly described taxa (Jayasiri et al., 2015; Index Fungorum, 2020). New species are established based on the guidelines provided by Jeewon and Hyde (2016).

### DNA Extraction, PCR Amplification, and Sequencing

Fungal genomic DNA was extracted from fruiting bodies using Forensic DNA Kit (Omega^®^, United States) following the manufacturer’s instructions and protocols outlined by Doilom et al. (2017) and Zeng et al. (2018). DNA was extracted from five duplicates of different fruiting bodies on the holotypic specimen of *Immotthia bambusae* (KUN-HKAS 112012) to allow verification of correct DNA sequence data. DNA amplification was performed by polymerase chain reaction (PCR). The primer pairs ITS5/ITS4 (White et al., 1990), LR0R/LR5 (Vilgalys and Hester, 1990), NS1/NS4 (White et al., 1990), and EF1-983F/EF1-2218R (Rehner, 2001) were used to amplify the PCR fragments of the internal transcribed spacers (ITS1-5.8S-ITS2), the 28S large subunit rDNA (LSU), the 18S small subunit rDNA (SSU), and the translation elongation factor 1-alpha (TEF1-α), respectively. PCR reactions were conducted in 25 μl total volume containing 2 μl of fungal genomic DNA template, 1 μl of each forward and reverse primer, 12.5 μl of 2 × Power Taq PCR Master Mix (mixture of EasyTaq^TM^ DNA Polymerase, dNTPs, and optimized buffer; Beijing BioTeke Corporation, China), and 8.5 μl of sterilized double-distilled water (ddH_2_O). The PCR thermal cycle profiles for ITS, LSU, SSU, and TEF1-α gene were set up following Jiang H. B. et al. (2020). PCR products were sent to TsingKe Biological Technology (Beijing) Co., Ltd., China, for PCR purification and sequencing.

### Alignment and Phylogenetic Analyses

The newly generated ITS, LSU, SSU and TEF1-α sequences of the new taxon were subjected to the BLASTn search tool^1^ for initial verification and search of reference taxa for further analyses. Similarity indices based on BLASTn search showed that five new strains are closely related to *Pseudocoleophoma* Kaz. Tanaka and K. Hiray (Dictyosporiaceae, Pleosporales). In order to investigate the phylogenetic status of the new taxa, a combined dataset of taxa including members of the Dictyosporiaceae was analyzed based on DNA sequence data available in recent publications (Iturrieta-González et al., 2018; Yang et al., 2018; Hyde et al., 2020a, b; Phukhamsakda et al., 2020). DNA sequences of representative taxa used are shown in Table 1. Individual DNA sequence alignments were initially performed *via* the online platform MAFFT v. 7.474^2^ (Katoh et al., 2019) and were improved manually using BioEdit v. 5.0.6 (Hall, 2001). Preliminary phylogenetic analyses of a concatenated LSU–SSU–TEF1-α–RPB2–ITS sequence matrix represented the relationships of *Immotthia* in Dictyosporiaceae with other families in Pleosporales (Supplementary Figure 3), and a concatenated ITS–LSU–TEF1-α sequence dataset (Supplementary Figure 1) was also analyzed by maximum-likelihood (ML) analysis *via* the web portal CIPRES Science Gateway v. 3.3 (Miller et al., 2010), with the help of the tool RAxML-HPC v.8 on XSEDE (8.2.12).

**TABLE 1:** Taxa names, strain numbers, and GenBank accession numbers of taxa used for the present phylogenetic analyses.

Taxa names	Strain/voucher no.	GenBank accession numbers			
		ITS	LSU	SSU	TEF1-α
***Aquadictyospora clematidis***	**MFLUCC 17-2080**	MT310592	MT214545	NG_070646	MT394727
***Aquadictyospora lignicola***	**MFLU 17-1422**	NR_157487	NG_064471	/	MF953164
***Aquaticheirospora lignicola***	**HKUCC10304**	AY864770	AY736378	AY736377	/
***Cheirosporium triseriale***	**HMAS 180703**	EU413953	EU413954	/	/
***Dendryphiella eucalyptorum***	**CBS 137987**	KJ869139	KJ869196	/	/
***Dendryphiella fasciculata***	**MFLUCC 17-1074**	NR_154044	NG_059177	/	/
***Dendryphiella paravinosa***	**CPC 26176**	NR_154012	NG_059137	/	/
***Dendryphiella phitsanulokensis***	**MFLUCC 17-2513**	NR_159827	NG_064502	NG_065729	/
***Dendryphiella variabilis***	**CBS 584.96**	NR_160584	LT963454	/	/
***Dictyocheirospora bannica***	**KH 332**	NR_154039	NG_059061	NG_064841	AB808489
***Dictyocheirospora garethjonesii***	**MFLUCC 16-0909**	KY320509	KY320514	/	/
*Dictyocheirospora garethjonesii*	DLUCC 0848	MF948623	MF948631	/	MF953166
*Dictyocheirospora indica*	MFLUCC 15-0056	MH381763	MH381772	MH381757	MH388817
***Dictyocheirospora pseudomusae***	**yone 234**	LC014550	AB807520	AB797230	AB808496
*Dictyocheirospora rotunda*	MFLU 18-1041	MH381764	MH381773	MH381758	MH388818
***Dictyocheirospora rotunda***	**MFLUCC 14-0293**	KU179099	KU179100	KU179101	/
***Dictyosporium appendiculatum***	**MFLUCC 17-2259**	NR_168192	MH376715	/	/
*Dictyosporium bulbosum*	yone 221	LC014544	AB807511	AB797221	AB808487
*Dictyosporium digitatum*	KH 401	LC014545	AB807515	AB797225	AB808491
*Dictyosporium digitatum*	yone 280	LC014547	AB807512	AB797222	AB808488
***Dictyosporium elegans***	**NBRC 32502**	DQ018087	DQ018100	DQ018079	/
***Dictyosporium meiosporum***	**MFLUCC 10-0131**	KP710944	KP710945	KP710946	/
***Dictyosporium olivaceosporum***	**KH 375**	LC014542	AB807514	AB797224	AB808490
***Dictyosporium tratense***	**MFLUCC 17-2052**	MH381767	MH381776	MH381761	MH388820
***Dictyosporium tubulatum***	**MFLUCC 15-0631**	MH381769	MH381778	/	MH388822
***Digitodesmium bambusicola***	**CBS 110279**	DQ018091	DQ018103	/	/
***Gregarithecium curvisporum***	**KT 922**	NR_154049	NG_059394	NG_061002	AB808523
***Immotthia bambusae***	**KUN-HKAS 112012AI**	MW489455	MW489450	MW489461	MW504646
***Immotthia bambusae***	**KUN-HKAS 112012AII**	MW489456	MW489451	MW489462	MW504647
***Immotthia bambusae***	**KUN-HKAS 112012B**	MW489457	MW489452	/	/
***Immotthia bambusae***	**KUN-HKAS 112012C**	MW489458	MW489453	MW489463	MW504648
***Immotthia bambusae***	**KUN-HKAS 112012D**	MW489459	MW489454	MW489464	MW504649
***Jalapriya pulchra***	**MFLUCC 15-0348**	KU179108	KU179109	KU179110	/
*Jalapriya pulchra*	MFLUCC 17-1683	MF948628	MF948636	/	MF953171
***Jalapriya toruloides***	**CBS 209.65**	DQ018093	DQ018104	DQ018081	/
*Neodendryphiella mali*	FMR 17003	LT993734	LT993735	/	/
***Neodendryphiella mali***	**FMR 16561**	LT906655	LT906657	/	/
***Neodendryphiella michoacanensis***	**FMR 16098**	NR_160583	NG_066395	/	/
***Neodendryphiella tarraconensis***	**FMR 16234**	NR_160582	NG_066394	/	/
*Periconia igniaria*	CBS 845.96	LC014586	AB807567	AB797277	AB808543
*Periconia igniaria*	CBS 379.86	LC014585	AB807566	AB797276	AB808542
***Pseudocoleophoma bauhiniae***	**MFLUCC 17-2586**	MK347736	MK347953	MK347844	MK360076
***Pseudocoleophoma calamagrostidis***	**KT 3284**	NR_154375	NG_059804	NG_061264	LC014614
*Pseudocoleophoma flavescens*	CBS 178.93	/	GU238075	GU238216	/
***Pseudocoleophoma polygonicola***	**KT 731**	NR_154274	NG_059393	NG_064848	AB808522
***Pseudocoleophoma rusci***	**MFLUCC 16-1444**	NR_170045	MT183514	NG_070346	/
***Pseudocoleophoma typhicola***	**MFLUCC 16-0123**	NR_154350	KX576656	/	/
***Pseudocoleophoma zingiberacearum***	**NCYUCC 19-0052**	MN615939	MN616753	/	MN629281
***Pseudoconiothyrium broussonetiae***	**CBS 145036**	NR_163377	NG_066331	/	/
***Pseudocyclothyriella clematidis*** *“Pseudocoleophoma clematidis”*	**MFLUCC 17-2177A**	MT310595	MT214548	MT226667	MT394730
***Pseudodictyosporium elegans***	**CBS 688.93**	NR_137148	NG_057743	NG_062684	/
*Pseudodictyosporium indicum*	CBS 471.95	DQ018097	/	/	/
***Pseudodictyosporium thailandica***	**MFLUCC 16-0029**	NR_154347	NG_059688	NG_063611	KX259526
***Pseudodictyosporium wauense***	**DLUCC 0801**	MF948622	MF948630	/	MF953165
*Vikalpa australiense*	HKUCC 8797	DQ018092	/	/	/

*CBS, Culture Collection of the Westerdijk Fungal Biodiversity Institute, Utrecht, Netherlands; CPC, Culture Collection of Pedro Crous, Netherlands; DLUCC, Dali University Culture collection, Yunnan, China; FMR, Facultat de Medicina, Universitat Rovira i Virgili, Reus, Spain; HKUCC, Hong Kong University Culture Collection, Hong Kong, China; HMAS, Herbarium Mycologicum Instituti Microbiologici, Academiae Sinicae, Beijing, China; KH, K. Hirayama; KUN-HKAS, Herbarium of Cryptogams Kunming Institute of Botany Academia Sinica, Yunnan, China; KT, K. Tanaka; MFLU, the Herbarium of Mae Fah Luang University, Chiang Rai, Thailand; MFLUCC, Mae Fah Luang University Culture Collection, Chiang Rai, Thailand; NBRC, Biological Resource Center, National Institute of Technology and Evaluation, Chiba, Japan; NCYUCC, National Chiayi University Culture Collection, Taiwan, China; yone, H. Yonezawa. The newly generated sequences and the ex-type strains are in black bold.*

ML analysis using the GAMMA model of nucleotide substitution was performed *via* the web portal CIPRES Science Gateway v.3.3 (Miller et al., 2010), with the help of the tool RAxML-HPC v.8 on XSEDE (8.2.12). The evolutionary model of nucleotide substitution for Bayesian inference (BI) analysis was selected independently for each locus using MrModeltest 2.3 (Nylander, 2008). GTR + I + G is the best fit for ITS and LSU loci under the Akaike information criterion (AIC), while the HKY + I substitution model is the best fit for SSU and TEF1-α loci. BI analysis was carried out by MrBayes v. 3.2.6 (Ronquist and Huelsenbeck, 2003). Markov chain Monte Carlo sampling (MCMC) was used to decide posterior probabilities (PP) (Rannala and Yang, 1996; Zhaxybayeva and Gogarten, 2002). Six simultaneous Markov chains were run for 1,000,000 generations and trees were sampled every 100th generation. The 0.15 “temperature” value was set in the MCMC heated chain. All sampled topologies beneath the asymptote (20%) were discarded as part of a burn-in procedure, and the remaining 8,000 trees were used for calculating posterior probabilities (PP) in the 50% majority rule consensus tree (when the split frequency was lower than 0.01). Tree topology of BI analysis is represented in Supplementary Figure 2.

Tree topologies generated in this study were visualized on FigTree v. 1.4.0^3^, and Microsoft Office PowerPoint 2016 (Microsoft Inc., United States) was used to edit and redraw the phylogram. New sequences generated from the present study are deposited in GenBank (Table 1). The final alignment and phylogram were submitted in TreeBASE^4^.

## Results

### Taxonomy

***Immotthia*** M. E. Barr, Mycotaxon 29: 504 (1987a), Figure 1.

**FIGURE 1:**
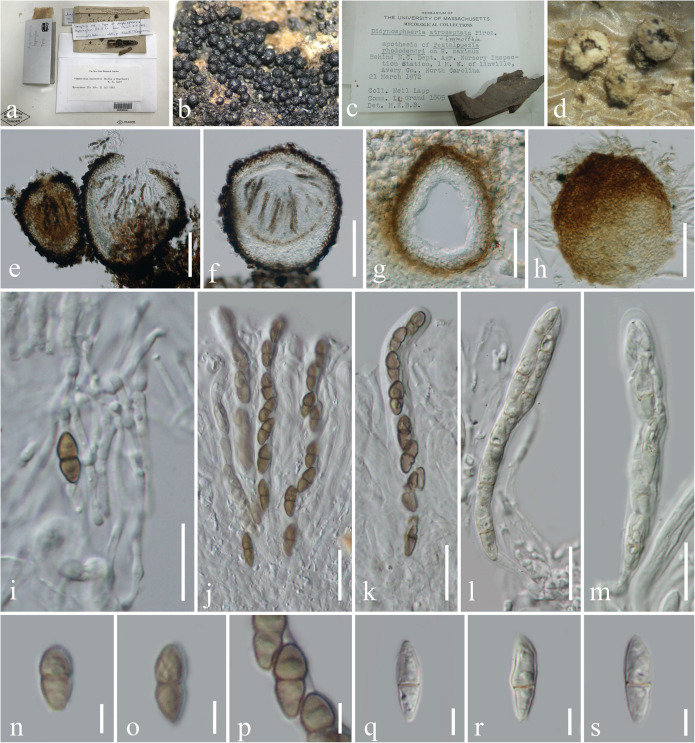
Morphological characteristics of *Immotthia*. **(c,d,g,h,j,k,q–s)** *Immotthia atroseptata* (DAOM 13900; holotype of *Didymosphaeria atroseptata*). **(a,b,e,f,i–k,n–p)** *Immotthia hypoxylon* (NY00830041, holotype of *Amphisphaeria hypoxylon*). **(a,c)** Herbarium label and specimens. **(b,d)** Appearance of ascomata on host substrates. **(e–g)** Section through ascomata. **(h)** Exterior of ascoma. **(i)** Pseudoparaphyses. **(j–m)** Asci. **(n–s)** Ascospores. Scale bars: **(e,f)** = 100 μm, **(g,h)** = 30 μm, **(i–m)** = 20 μm, and **(n–s)** = 5 μm.

*Index Fungorum Number*: IF 25106

*Facesoffungi Number*: FoF 08362

*Hyperparasitic* on *Annulohypoxylon*, *Hypoxylon*, and *Pestalopezia*, or *saprobic* on decorticated wood. *Sexual morph*: *Hypostroma* dark brown to black, crust under ascomata, patch-like, composed of thick-walled cells of *textura angularis*, covering the surface of host stromata, difficult to distinguished from the peridium of ascomata, sometimes forming dark brown hyphae at the lowest layer penetrated with the host of *Hypoxylon* which was interpreted to belong to the *Hypoxylon* host. *Ascomata* dark brown to black, subglobose to obpyriform, or inequilateral, gregarious to densely aggregated, superficial, uniloculate, or carbonaceous when dry, glabrous, surface rough with protruding cells, arising from a large hypostroma, usually with a pore-like, inconspicuous ostiole or minute papilla. *Peridium* thin- to thick-walled, of unequal thickness, composed of several layers, inner layers comprising hyaline to dark brown, pseudoparenchymatous cells, of *textura angularis*, outer layers composed of thick, dark brown to black cells, arranged in a *textura angularis*. *Hamathecium* composed of dense, septate, branched, anastomosing, cellular pseudoparaphyses embedded in a gelatinous matrix. *Asci* (4–) (6–)–8-spored, bitunicate, fissitunicate, cylindrical or cylindric-clavate, subsessile to short-pedicellate with obtuse knob-like or furcate pedicel, apically rounded with a well-developed ocular chamber. *Ascospores* overlapping 1–2-seriate, pale yellowish to brown, or reddish brown, ellipsoidal to fusiform, with rounded ends, 1-septate, asymmetrical, with the upper cell slightly larger than the lower cell, slightly constricted at the septum, smooth- to rough-walled, verrucose. *Asexual morph*: Coelomycetous, chaetophoma-, coniothyrium-, microsphaeropsis-, or pyrenochaeta-like, associated with the sexual morph on natural substrate. *Conidiomata* pycindial, similar to ascomata but differ in having a smaller size, black, carbonaceous, immersed to erumpent, becoming superficial, globose to obpyriform, uni- to multiloculate, glabrous, with indistinct ostiolate. *Pycnidial wall* thin- to thick-walled, composed of several layers of brown to dark brown pseudoparenchymatous cells, of *textura angularis*. *Conidiophores* reduced to conidiogenous cells. *Conidiogenous cells* enteroblastic, phialidic, discrete, determinate, ampulliform, or cylindric, smooth, hyaline, with minute collarette and conspicuous periclinal thickening. *Conidia* ellipsoidal, rounded at both ends, one-celled, at first hyaline, becoming brown at maturity, smooth-walled or finely verrucose (adapted from Jaklitsch et al., 2002; Akulov and Hayova, 2016; Hyde et al., 2017; Doilom et al., 2018).

*Type Species*: *Immotthia hypoxylon* (Ellis and Everh.) M. E. Barr, Mycotaxon 29: 504 (1987a).

≡ *Amphisphaeria hypoxylon* Ellis and Everh., J. Mycol. 2(4): 41 (1886).

*Current Name*: *Immotthia atrograna* (Cooke and Ellis) M. E. Barr (1993), Mycotaxon 46: 71.

≡ *Sphaeria atrograna* Cooke and Ellis, Grevillea 8(no. 45): 15 (1879).

*Life Mode and Known Distribution*: Hyperparasitic on *Annulohypoxylon*, *Hypoxylon*, and *Pestalopezia* on various host substrates and saprobic on decaying wood. *Immotthia* is presently known from Austria, Belgium, China, France, Lithuania, Norway, Poland, Puerto Rico, Russia, Sweden, Switzerland, Ukraine, United States, and Venezuela (Pirozynski, 1973; Jaklitsch et al., 2002; Akulov and Hayova, 2016; Hyde et al., 2017; Doilom et al., 2018; Farr and Rossman, 2020).

*Notes*: The phylogenetic affinities of *Immotthia* have never been investigated in previous studies, and the genus was accommodated in different families by different authors based only on morphological characteristics (Barr, 1987a, 2002; Jaklitsch et al., 2002; Akulov and Hayova, 2016; Hyde et al., 2017; Doilom et al., 2018). In the present study, *Immotthia* is phylogenetically close to *Pseudocoleophoma* in Dictyosporiaceae. The sexual morph of *Immotthia* differs from *Pseudocoleophoma* in morphology and habitat. *Immotthia* forms dense, superficial ascomata on hypostoma, with cylindrical to cylindric-clavate asci, ellipsoidal to fusiform, light brown to reddish brown, asymmetrical ascospores, lacking mucilaginous sheath and usually found as hyperparasites on hypoxylon-like stroma. On the other hand, *Pseudocoleophoma* forms scattered or in groups, immersed to erumpent ascomata, with cylindrical to clavate asci, fusiform, hyaline ascospores, surrounded by mucilaginous sheath, and mostly found as saprobes in a terrestrial environment (Tanaka et al., 2015; Jayasiri et al., 2019; Tennakoon et al., 2019; Li et al., 2020). The asexual morph of *Immotthia* differs from *Pseudocoleophoma* in having multiloculate, carbonaceous conidiomata and ellipsoidal, brown, aseptate conidia (Hyde et al., 2017), whereas *Pseudocoleophoma* has uniloculate conidiomata and cylindrical to subcylindrical or fusiform, hyaline, 0–1-septate conidia.

**FIGURE 2:**
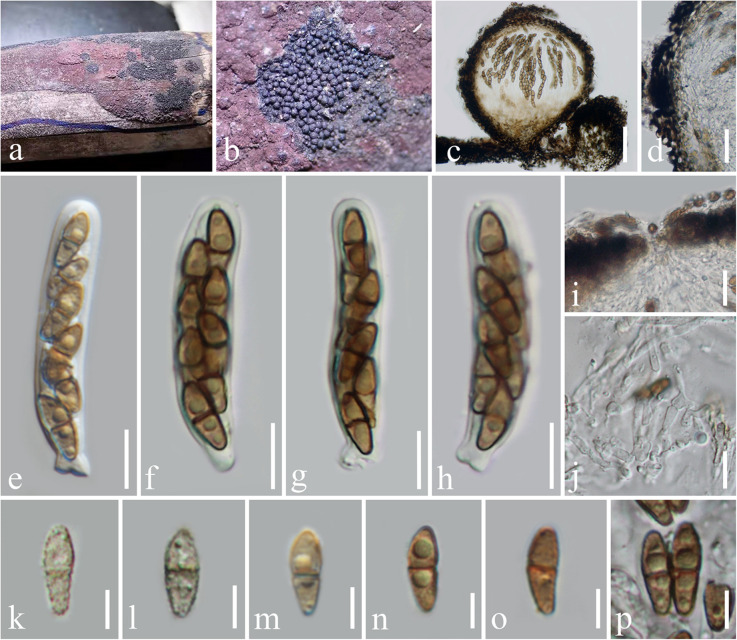
*Immotthia bambusae* (KUN-HKAS 112012, holotype). **(a,b)** Appearance of hypostromata on *Hypoxylon* sp. associated with dead bamboo culms. **(c)** Vertical section of ascoma. **(d)** Peridium. **(e–h)** Asci. **(i)** Pore-like ostiole. **(j)** Pseudoparaphyses. **(k–p)** Ascospores. Scale bars: **(c)** = 50 μm, **(d)** = 20 μm, **(i)** = 15 μm, **(e–h,j)** = 10 μm, and **(k–p)** = 5 μm.

***Immotthia bambusae*** H. B. Jiang and Phookamsak, **sp. nov.,** Figure 2.

*Index Fungorum Number*: IF 557242

*Facesoffungi Number*: FoF 09538

*Etymology*: The specific epithet “*bambusae*” refers to the host, bamboo, of which the new species was collected.

*Holotype*: KUN-HKAS 112012

*Hyperparasitic* on *Hypoxylon* sp. on dead bamboo culms. *Sexual morph*: *Hypostroma* effuse, black, with numerous, superficial ascomata, composed of thin layered, of blackened, pseudoparenchymatous cells of *textura angularis*. *Ascomata* 130–210 μm high, 150–220 μm diam., dark brown to black, scattered, gregarious, globose to subglobose, or obpyriform, arising from the hypostroma, carbonaceous, brittle when dry, easily dispersed, uniloculate, glabrous, rough-walled, with a pore-like, inconspicuous ostiole. *Peridium* 15–30 μm wide, of unequal thickness, slightly thick at the base, composed of several layers of pseudoparenchymatous cells, outer layers composed of thick-walled, blackened cells, arranged in *textura angularis* to *textura globulosa*, inner layers composed of flattened, brown cells, of *textura angularis* to *textura prismatica*, paler toward the inner layers. *Hamathecium* composed of dense, 1.5–2 μm broad, cellular pseudoparaphyses, septate, branched, anastomosing among the asci, embedded in gelatinous matrix. *Asci* 45–60(–62) × 7.5–10 μm (*x̄* = 53 × 9 μm, *n* = 15), (6–)8-spored, bitunicate, fissitunicate, cylindrical, subsessile, with knob-like or furcate pedicel, apically rounded with well-developed ocular chamber. *Ascospores* 9–11 × 3–4.5 μm (*x̄* = 10 × 3.7 μm, *n* = 20), overlapping 1–2-seriate, light brown to brown, ellipsoidal to fusiform, with rounded ends, lower cell slightly longer and narrower than the upper, 1-septate, rough-walled, finely verrucose, initially presented small to large guttules, disappeared when mature, without sheath. *Asexual morph*: Undetermined.

*Material Examined*: Thailand, Loei Province, a wild bamboo forest (17°15′44.68″N, 101°8′39.32″E, altitude 364.09 m), on *Hypoxylon* sp. associated with dead culms of bamboo, February 25, 2020, H. B. Jiang and R. Phookamsak, BBL07-2-1 (KUN-HKAS 112012, holotype; HMAS 249866, isotype).

*Life Mode and Known Distribution*: Hyperparasitic on *Hypoxylon* sp. associated with bamboo culms (Thailand).

*Notes*: The type specimens of all *Immotthia* species were compared; *I. bambusae* shows the most similarity to *I. hypoxylon*, a synonym of *I. atrograna*. However, the novel taxon differs from *I. atrograna* (= *I. hypoxylon*) in having a smaller size of ascomata, asci, and ascospores. The lower cell of ascospores of *I. bambusae* is longer and narrower than the upper cell, while in *I. atrograna* (= *I. hypoxylon*), the upper cell of ascospores is larger than the lower cell. *Immotthia bambusae* was collected on the stromata of *Hypoxylon* associated with bamboo in Thailand (tropical region), whereas the type specimen of *I. hypoxylon* was collected from *Hypoxylon truncatum* colonizing dead canes of *Rosa* in Louisiana, United States (subtropical region). The other different features between *I. bambusae* and the other *Immotthia* species are provided in Table 2. Therefore, *I. bambusae* is introduced as a new species in this study based on both morphology and multigene phylogeny.

**TABLE 2:** Synopsis of *Immotthia* species based on type studies (*I. hypoxylon* is listed separately from *I. atrograna* in this study for a better understanding).

Species name	Ascomata	Asci	Ascospores	Asexual morph	Host occurrences	References
*Immotthia atrograna* (= *I. hypoxylon*)	120–270 μm diam., 150–310 μm high, carbonaceous, glabrous, densely aggregated in large groups or loosely scattered, globose to obpyriform, with pore-like, cream to reddish brown, 30–50 diam., inconspicuous ostiole	60–86 × 6–10 μm, 8-spored, oblong to cylindrical, shortly pedicellate with knob-like or obtuse pedicel	(8–)9–14(–18) × 5–6 μm, 1-seriate, yellowish brown to reddish brown, ellipsoid to biconical, often upper cell slightly shorter and broader than the lower, ends rounded to subacute, 1-septate, constricted at the septum, smooth to slightly verrucose	*Coniothyrium parasitans*	Aceri-Fraxinetum, *Carya olivaeformis*, *Carya* sp., *Hypoxylon rubigmosum*, *H. rubiginosum* on *Fraxinus excelsior*, *H. rubigmosum* on *Fraxinus* sp., *H. rubigmosum* on *Salix alba*, *Hypoxylon* sp., *Hypoxylon* sp. on *Acer pseudoplatanus*, *Hypoxylon* sp. on *Fraxinus excelsior*, *Hypoxylon* sp. on *Fraxinus* sp., *Liquidambar* sp., *Robinia* sp.	Jaklitsch et al., 2002
*Immotthia atroseptata*	170–250 μm diam., 190–350 μm high, simple or aggregated, globose to obpyriform, with conical or truncate apex perforated by a small, inconspicuous ostiole	62–95 × 8–11 μm, 8-spored, clavate, with a short furcate pedicel	13–17.5 × 4.5–6 μm, 1–2-seriate, brown to reddish brown, ellipsoidal to fusiform with both rounded ends, the upper cell slightly larger than the lower, 1-septate, constricted at the septum, smooth-walled	Undetermined	Apothecia of *Pestalopezia rhododendri* on fallen leaves of *Rhododendron maximum*	Pirozynski, 1973; Doilom et al., 2018
*Immotthia bambusae*	150–220 μm diam., 130–210 μm high, carbonaceous, scattered, rarely solitary, gregarious, globose to subglobose, with rounded apex with a pore-like, inconspicuous ostiole	45–60(–62) × 7.5–10 μm, 6–8-spored, cylindrical, sessile to subsessile, with knob-like or furcate ends	9–11 × 2.8–4.5 μm, 1–2-seriate, light brown to brown, ellipsoidal to fusiform, with rounded ends, lower cell slightly longer and narrower than the upper, 1-septate, finely verrucose	Undetermined	*Hypoxylon* sp. on bamboo	This study
*Immotthia hypoxylon*	120–280 μm diam., 170–290 μm high, carbonaceous, glabrous, scattered to clustered, gregarious, subglobose to obpyriform, with pore-like ostiole	(67–)70–90(–108) × 8–11 μm, 8-spored, cylindrical, shortly pedicellate with a knob-like or obtuse end	10–13(–15) × 4–6 μm, 1-seriate, brown to reddish brown, ellipsoidal to fusiform, with round ends, 1-septate, slightly constricted at the septum, rough-walled	*Coniothyrium parasitans*	*Hypoxylon investiens* on *Quercus* sp., *Hypoxylon* sp. on *Fraxinus* sp., *H. truncatum* on dead canes of *Rosa* sp., *Tilia* sp., wood	Hyde et al., 2017

In this study, we sequenced five different fruiting bodies and DNA sequence similarity (ITS regions) revealed that they have identical nucleotides (100% similarity). Even our phylogeny depicts a close relationship (100% support) among these five strains. This ensures the correctness of the new generated sequences from the direct DNA extraction of fruiting bodies.

***Pseudocyclothyriella*** Phukhams. and Phookamsak, **gen. nov.**

*Index Fungorum Number*: IF 557441

*Facesoffungi Number*: FoF 09539

*Etymology*: The generic epithet “*Pseudocyclothyriella*” refers to the resemblance of conidial morphology of the new genus to the genus *Cyclothyriella*.

*Saprobic* on *Clematis vitalba* (Ranunculaceae). *Sexual morph*: Undetermined. *Asexual morph*: *Conidiomata* pycnidial, solitary to gregarious, uniloculate, immersed to erumpent, laterally becoming superficial, visible as black, shiny on host, subglobose to subconical, coriaceous, subcoriaceous at the outer layers, glabrous, dark brown to black, ostiolate, papillate. *Ostioles* central, ovoid, with minute papilla, filled with hyaline periphyses. *Pycnidial wall* thick-walled of equal thickness, composed of multilayered scleroplectenchymatous cells, outer layer composed of several layers of thick-walled, dark brown to black cells of *textura angularis* to *textura globulosa*, inner layer composed of hyaline to pale brown cells, bearing conidiogenous cells. *Conidiophores* reduced to conidiogenous cells. *Conidiogenous cells* holoblastic, phialidic, determinate, discrete, cylindrical to subcylindrical, or ampulliform, hyaline, aseptate, smooth-walled, arising from the inner layers of conidioma. *Conidia* oval to oblong, hyaline to yellowish brown, slightly curved toward the ends, aseptate, smooth-walled.

*Type Species*: *Pseudocyclothyriella clematidis* (Phukhams. and K. D. Hyde) Phukhams. and Phookamsak.

*Life Mode and Known Distribution*: *Pseudocyclothyriella* is reported as a saprobe on *Clematis vitalba* (Ranunculaceae). The genus is presently known from Italy (Phukhamsakda et al., 2020).

*Notes*: Based on morphological distinctiveness and multigene phylogenetic analyses, a monotypic genus *Pseudocyclothyriella* is introduced herein to accommodate a single coelomycetous species, *P. clematidis* which was previously described as *Pseudocoleophoma clematidis* by Phukhamsakda et al. (2020). *Pseudocyclothyriella* formed an independent clade basal to *Immotthia* and *Pseudocoleophoma* with strong statistical supports (92% ML, 0.98 PP). *Pseudocyclothyriella* is similar to *Cyclothyriella* in having cylindrical, oblong to ellipsoid, aseptate, hyaline to pigmented conidia (Jaklitsch and Voglmayr, 2016). However, *Pseudocyclothyriella* can be distinguished from *Cyclothyriella* based on the conidiomatal characteristics and phylogenetic evidence. *Pseudocyclothyriella* is characterized by solitary to gregarious, immersed to erumpent, black, shiny, subglobose to subconical conidiomata, with oval, papilla, ostiolar canal, and pycnidial wall composed of thick-walled, scleroplectenchymatous cells. On the other hand, *Cyclothyriella* has black, more or less globose pycnidia, clustered in valsoid configuration, with brightly colored, disc-like ostiole, and pycnidial wall composed of pseudoparenchymatous cells (Jaklitsch and Voglmayr, 2016). *Cyclothyriella* belongs to its own family Cyclothyriellaceae, whereas *Pseudocyclothyriella* belongs to Dictyosporiaceae.

*Pseudocoleophoma* differs from *Pseudocyclothyriella* in pycnidial wall composed of thin-walled, brown to dark brown pseudoparenchymatous cells and oblong, cylindrical to subcylindrical, or rod-shaped, 0–1-septate, smooth-walled conidia (Tanaka et al., 2015; Jayasiri et al., 2019; Tennakoon et al., 2019; Li et al., 2020). *Pseudocyclothyriella* is morphologically similar to a presumably coniothyrium-like asexual morph of *Immotthia* in having black, carbonaceous pycnidia, and pigmented, aseptate, smooth-walled conidia (Hyde et al., 2017). However, *Immotthia* forms pycnidia in groups, or multiloculate conidiostromata associated with the ascomata of *Immotthia* on natural host substrates and has ellipsoidal conidia as well as having hyperparasitic life mode (Hyde et al., 2017), while *Pseudocyclothyriella* forms solitary to gregarious, uniloculate pycidia and has oval to oblong conidia as well as having saprobic life mode (Phukhamsakda et al., 2020). The sexual morph of *Pseudocyclothyriella* is undetermined; thus, the sexual morphologies of *Pseudocyclothyriella* and *Immotthia* could not be compared.

**FIGURE 3:**
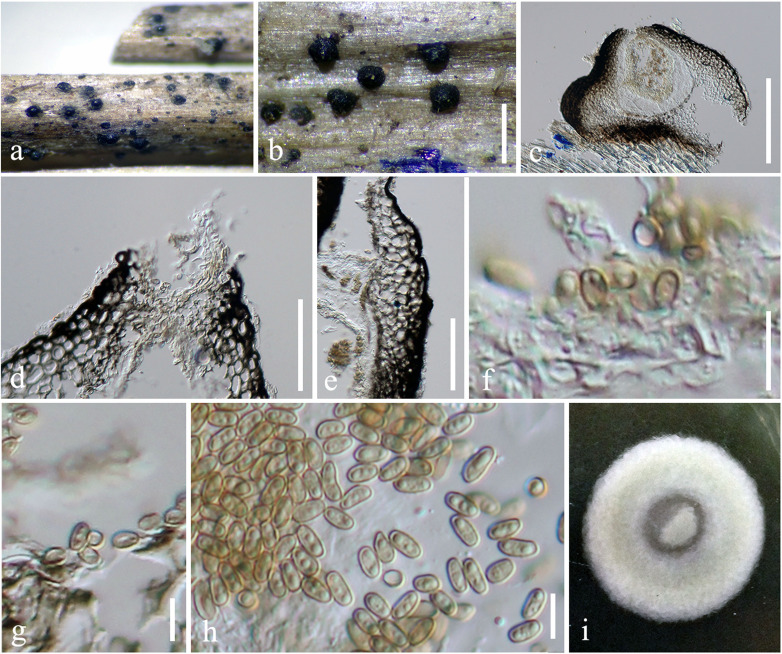
*Pseudocyclothyriella clematidis* (MFLU 16-0280, holotype). **(a,b)** Appearance of pycnidial conidiomata on host substrates. **(c)** Vertical section of conidioma. **(d)** Ostiole. **(e)** Pycnidial wall. **(f,g)** Conidiogenous cells with attached conidia. **(h)** Conidia. **(i)** Culture colony on MEA (upper view). Scale bars: **(b)** = 500 μm, **(c)** = 200 μm, **(d,e)** = 20 μm, and **(f–h)** = 5 μm.

***Pseudocyclothyriella clematidis*** (Phukhams. and K. D. Hyde) Phukhams. and Phookamsak, **comb. nov.,** Figure 3

*Index Fungorum Number*: IF 557442

*Facesoffungi Number*: FoF 09540

≡ *Pseudocoleophoma clematidis* Phukhams. and K. D. Hyde, in Phukhamsakda et al., Fungal Divers (102): 21 (2020).

*Holotype Details*: Italy, Arezzo Province, Badia Tega—Ortignano Raggiolo, on dead aerial branch of *Clematis vitalba*, March 9, 2013, E. Camporesi, IT 1110 (MFLU 16-0280), ex-type living culture, MFLUCC 17-2177.

*Description and Illustration*: See Phukhamsakda et al. (2020).

*Ecology and Known Distribution*: As a saprobe, *Pseudocyclothyriella clematidis* has contributions in the cycle of the material. To date, the species is just reported in Italy (Phukhamsakda et al., 2020).

*Notes*: *Pseudocyclothyriella clematidis* was previously treated in *Pseudocoleophoma* based on phylogenetic evidence (Phukhamsakda et al., 2020). *Pseudocyclothyriella clematidis* differs from the other *Pseudocoleophoma* species in having yellowish brown, oval to oblong, aseptate conidia (Phukhamsakda et al., 2020). A morphological comparison of *P. clematidis* with other *Pseudocoleophoma* species is provided in Table 3. The two strains of *Pseudocyclothyriella clematidis* (MFLU 16-0280 and MFLUCC 17-2177A) formed a strongly supported clade in this study. Phukhamsakda et al. (2020) introduced *Pseudocoleophoma clematidis* based on phylogenetic evidence and reported the species was poorly supported in a subclade in between the main clade of *Pseudocoleophoma* and *P. typhicola* (MFLUCC 16-0123). However, in our multigene phylogeny, we recovered the same taxon as basal to *Pseudoconiothyrium broussonetiae*.

**TABLE 3:** Synopsis of morphological characteristics of *Pseudocoleophoma* and *Pseudocyclothyriella.*

Species name	Sexual morph			Asexual morph			References
	Ascomata	Asci	Ascospores	Conidiomata	Conidiogenous cells	Conidia	
*Pseudocoleophoma bauhiniae*	100–120 × 125–145 μm, solitary or scattered, dark brown, subglobose to obpyriform, coriaceous	65–80 × 5–8 μm, clavate to cylindric-clavate	17–20 × 3.5–4.5 μm, hyaline, cylindric-fusiform, 1–3-septate, without sheath	130–150 × 90–115 μm, immersed to superficial, globose to subglobose, glabrous, multiloculate, ostiolate	2.5–5.5 × 2–3 μm, phialidic, hyaline, doliiform to lageniform	7.5–11 × 2–3 μm, hyaline, oblong to ellipsoidal, with rounded or obtuse ends, aseptate	Jayasiri et al., 2019
*P. calamagrostidis*	160–220 × 140–200 μm, scattered, dark brown, globose to depressed globose	62.5–80 × 7.5–10 μm, cylindrical	16–19 × 3–4.5 μm, hyaline, narrowly fusiform, 1-septate, with sheath	250–500 × 220–300 μm, immersed to erumpent, depressed globose, glabrous, uniloculate, ostiolate	5–9 × 2–4 μm, phialidic, hyaline, doliiform to subglobose	6–10 × 2–2.5 μm, hyaline, cylindrical, aseptate	Tanaka et al., 2015
*P. flavescens*	N/A	N/A	N/A	20–140 μm diam., solitary or confluent, globose, glabrous or covered by hyphae	4–6 × 3–6 μm, globose to doliiform	4–7 × 2–3.5 μm, hyaline, oblong to ellipsoidal with two very large polar guttules, aseptate	De Gruyter et al., 1993; Li et al., 2020
*P. polygonicola*	280–350 × 230–310 μm, scattered to 2–4-gregarious, brown to dark brown, globose to subglobose	74–90 × 9–12.5 μm, cylindrical to clavate	19–23 × 4–6 μm, hyaline, fusiform, 1-septate, with sheath	170–250 μm diam., superficial, ampulliform, glabrous, uniloculate, ostiolate	7–17 × 3.5–5 μm, phialidic, hyaline, doliiform to lageniform	11.5–18 × 3–4.5 μm, hyaline cylindrical, aseptate	Tanaka et al., 2015
*P. rusci*	N/A	N/A	N/A	130–200 × 250–330 μm, deeply immersed, globose, subglobose or ovoid, glabrous, uniloculate, ostiolate	4–9 × 3–7 μm, enteroblastic, phialidic, hyaline, doliiform, ampulliform to subcylindrical	8–14 × 3–6 μm, hyaline, cylindrical to subcylindrical or fusiform, aseptate	Li et al., 2020
*P. typhicola*	N/A	N/A	N/A	60–100 × 140–150 μm, semi-erumpent, subglobose, glabrous, uniloculate	2–5 × 2–5 μm, enteroblastic, hyaline, subcylindrical	9–11 × 2–3 μm, hyaline, oblong to cylindrical, 1-euseptate	Hyde et al., 2016
*P. zingiberacearum*	N/A	N/A	N/A	200–220 × 110–150 μm, immersed, depressed globose, glabrous, multiloculate, non-ostiolate	1.5–2.5 × 1–1.5 μm, phialidic, hyaline, doliiform to lageniform	12–14 × 2–3 μm, hyaline, oblong to ellipsoidal, aseptate	Tennakoon et al., 2019
*Pseudocyclothyriella clematidis* (≡ *Pseudocoleophoma clematidis*)	N/A	N/A	N/A	130–150 × 100–130 μm, immersed, globose to subglobose, glabrous, uniloculate, ostiolate	2–4 × 1.5–4 μm, holoblastic, phialidic, hyaline, cylindrical to subcylindrical	5–8 × 2–4 μm, yellowish brown, oval, aseptate	Phukhamsakda et al., 2020; This study

**FIGURE 4:**
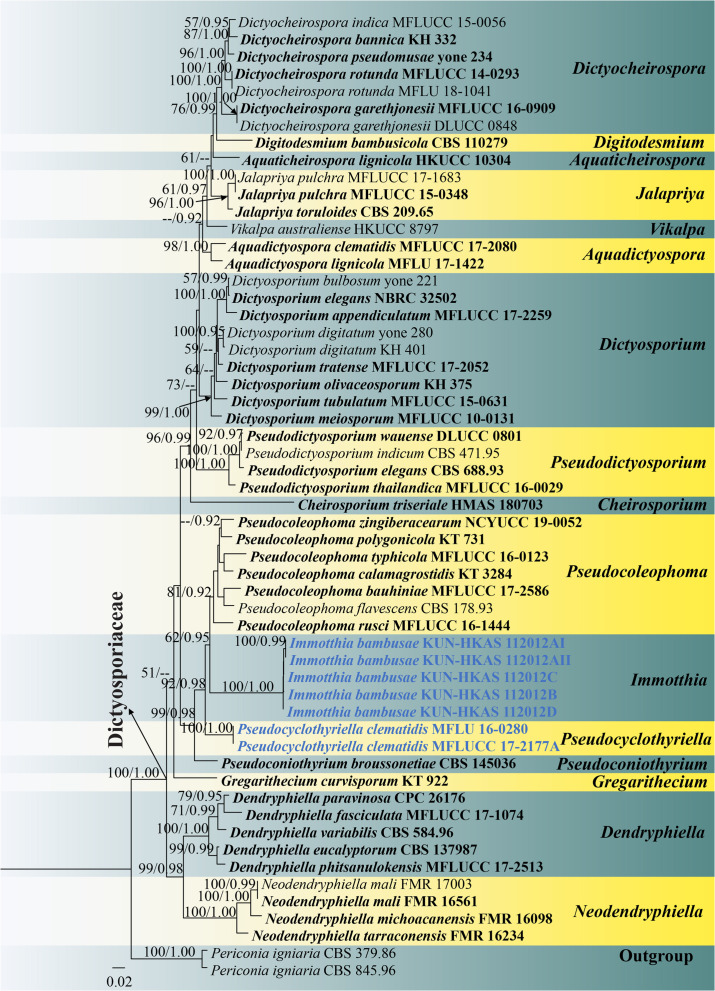
RAxML tree based on a combined ITS, LSU, SSU, and TEF1-α sequence matrix represented the phylogenetic relationships of taxa in Dictyosporiaceae. The tree is rooted to *Periconia igniaria* (CBS 845.96 and CBS 379.86). Bootstrap support values for ML equal to or greater than 50% and the Bayesian posterior probabilities equal to or higher than 0.90 PP are indicated above the nodes as ML/PP. Ex-type strains are in black bold and the new species and new combinations are indicated in blue bold.

Crous et al. (2019) mentioned that the closest hits of *P. broussonetiae* using the ITS sequence had highest similarity to *Pseudocoleophoma typhicola* (MFLUCC 16-0123). However, *P. typhicola* is morphologically different from *P. broussonetiae*, but is typical of *Pseudocoleophoma*. We rechecked the BLASTn search result based on ITS and LSU sequences of *P. typhicola* (MFLUCC 16-0123) available in GenBank and noted that the DNA sequences from the ITS regions of *P. typhicola* are similar to the endophytic Pleosporales sp. isolate MBD_4078 (MK595603) and the uncultured fungus clone ITS_S7_clon2 (HQ873356) with 90.50% similarities and matches with *P. broussonetiae* strain CBS 145036 (NR_163377) with 90.23% similarity which is far away from *Pseudocoleophoma*. On the other hand, the BLASTn search result based on LSU sequence showed that the species is closely related with *Pseudocoleophoma*, and hence, these may be erroneous. In this study, we therefore excluded the ITS sequence of *P. typhicola* from our aligned sequence dataset and the phylogenetic results showed that *P. typhicola* clusters within *Pseudocoleophoma* (Figure 4). However, *P. typhicola* needs to be resequenced as well as obtaining more reliable genes (only ITS and LSU are available in GenBank) for a better phylogenetic resolution is needed.

### Phylogenetic Analyses

The closest query cover identification of five new strains (KUN-HKAS 112012AI, KUN-HKAS 112012AII, KUN-HKAS 112012B, KUN-HKAS 112012C, and KUN-HKAS 112012D) on BLASTn search tool indicated that our new *Immotthia* taxon is similar to *Pseudocoleophoma rusci* (MFLUCC 16-1444), *P. calamagrostidis* (KT 3284), *P. polygonicola* (KT 731), and *Dictyocheirospora pseudomusae* (yone 234) when DNA sequences (ITS, LSU, SSU, and TEF1-α) were compared.

Phylogenetic analyses of a combined ITS, LSU, SSU, and TEF1-α sequence matrix were performed based on 56 strains of taxa in Dictyosporiaceae and two strains of *Periconia igniaria* (CBS 845.96 and CBS 379.86) as outgroup. This dataset consists of 3,397 total characters including gaps (ITS: 1–585 bp, LSU: 586–1,445 bp, SSU: 1,446–2,473 bp, TEF1-α: 2,474–3,397 bp). The best scoring ML tree was selected to represent the phylogenetic relationships of the new taxon with other representative taxa in Dictyosporiaceae (Figure 4), with the final ML optimization likelihood value of −17,968.180137 (ln). All free model parameters were estimated by GTRGAMMA model, with 1,026 distinct alignment patterns and 33.65% of undetermined characters or gaps. Estimated base frequencies were as follows: *A* = 0.240488, *C* = 0.242841, *G* = 0.269410, and *T* = 0.247261, with substitution rates AC = 1.638750, AG = 3.305690, AT = 2.424291, CG = 0.898018, CT = 8.545431, and GT = 1.000000. The gamma distribution shape parameter alpha = 0.169318 and the Tree-Length = 1.822628. The final average standard deviation of split frequencies at the end of total MCMC generations was calculated as 0.009979 in BI analysis.

Preliminary phylogenetic analysis based on a concatenated LSU–SSU–TEF1-α–RPB2–ITS sequence matrix of representative families in Pleosporales depicts that *Immotthia* belongs to the family Dictyosporiaceae (Supplementary Figure 3). The phylograms from ML and BI analyses (Figure 4 and Supplementary Figure 2) of a concatenated ITS–LSU–SSU–TEF1-α sequence matrix were similar in overall topologies and were also similar to the tree topology of a concatenated ITS–LSU–TEF1-α sequence dataset (Supplementary Figure 1). Our five new strains of *I. bambusae* constitute a strongly supported independent subclade basal to *Pseudocoleophoma* (62% ML, 0.95 PP). Two strains of *Pseudocyclothyriella clematidis* (≡ *Pseudocoleophoma clematidis*, MFLU 16-0280 and MFLUCC 17-2177A) formed an independent subclade basal to *Immotthia* with strong statistical supports (92% ML, 0.98 PP). Based on current phylogenetic status and morphological distinctiveness compared with the other *Pseudocoleophoma* species, *Pseudocoleophoma clematidis* is transferred to the novel genus *Pseudocyclothyriella* as *P. clematidis*.

## Discussion

An updated taxonomic treatment of bambusicolous fungi has been conducted since the last decade by Japanese mycologists (Tanaka et al., 2009, 2015) and followed by many Asian mycologists (Liu et al., 2011, 2012, 2014, 2015; Dai et al., 2012, 2014a, 2014b, 2014c, 2017, 2018, 2019; Phookamsak et al., 2014, 2015, 2018; Adamčík et al., 2015; Ariyawansa et al., 2015; Senanayake et al., 2015, 2020a; Jiang et al., 2018, 2019a,b, 2019c; Sommai et al., 2019; Yang et al., 2019a,b; Jiang H. B. et al., 2020; Jiang N. et al., 2020; Hyde et al., 2020a, b; Monkai et al., 2020; Sun et al., 2020; Tang et al., 2020; Xie et al., 2020; Zhang et al., 2020). To date, more than 1,300 bambusicolous fungi have been reported consisting of 150 basidiomycetes and 1,150 ascomycetes. However, the taxonomic placements of many described species have yet to be verified based on DNA sequence phylogeny (Dai et al., 2018).

In this study, we collected a fungus associated with *Hypoxylon* stromata on bamboo from northeastern Thailand. Based on morphological examination comparable with the type specimens, the species is identified as a typical *Immotthia* but largely different to warrant the establishment of a new species. Thus, we introduce a novel species *I. bambusae* and this is also the first report of *Immotthia* associated with *Hypoxylon* stromata on bamboo in Thailand. Furthermore, it is the first time that DNA sequence data of *Immotthia* are obtained and its phylogenetic affinity within the Dictyosporiaceae was investigated. In addition, a novel genus *Pseudocyclothyriella* is introduced as a monotypic genus to accommodate *P. clematis* during the phylogenetic investigation of *Immotthia*.

Multigene phylogenetic analyses showed that *Immotthia* formed a well-resolved clade within Dictyosporiaceae in all analyses (Figure 4 and Supplementary Figures 1–3). The genus clustered with *Pseudocoleophoma* in all analyses with significant support in BI analysis (0.95 PP; Figure 4), but low support in ML analysis (62% ML; Figure 4). However, *Immotthia* is also morphologically different from *Pseudocoleophoma* (see notes under generic description).

*Immotthia* is widely distributed from tropical to temperate regions including Austria, Belgium, China, France, Lithuania, Norway, Poland, Puerto Rico, Russia, Sweden, Switzerland, Ukraine, United States, and Venezuela (Pirozynski, 1973; Jaklitsch et al., 2002; Akulov and Hayova, 2016; Hyde et al., 2017; Doilom et al., 2018; Farr and Rossman, 2020). *Immotthia* does not seem to exhibit a hyperparasitic lifestyle on *Hypoxylon*, but species of this genus were also reported as saprobes on various decayed hardwoods (Jaklitsch et al., 2002). *Immotthia bambusae* did not germinate on potato dextrose agar (PDA) medium, suggesting that the species has possibly an obligate parasitic life mode, which is in agreement with Jaklitsch et al. (2002).

Jaklitsch et al. (2002) treated the type species of *Immotthia*, *I. hypoxylon*, as a synonym of *I. atrograna* after they examined the type materials of these two species. They found that the basionym of both *I. hypoxylon* and *I. atrograna* shared similar size range of ascomata, asci, and ascospores and it does not show any convincing difference on the ascomata, although these two species occurred on different hosts and habitats (Jaklitsch et al., 2002). Hyde et al. (2017) re-examined the type material of *I. hypoxylon* and compared it with other collections from North America and reported similar morphology, but the latter have larger ascomata and shorter asci than the type (Hyde et al., 2017). A comparison of the type examination between *I. hypoxylon* (Hyde et al., 2017) and *I. atrograna* (Jaklitsch et al., 2002) shows that *I. atrograna* has a larger ascomata, smaller asci, and overlapped size range of ascospores. However, these two species still lack DNA sequence data, and hence, their taxonomic status with regards to whether they are conspecific warrants further investigation. Herein, we follow the treatment of Jaklitsch et al. (2002) until the epitypes of these two species are designated and their taxonomy is revisited.

Dictyosporiaceae comprises 17 genera, including *Immotthia* and *Pseudocyclothyriella*. Asexual morph of *Immotthia* is recognized as chaetophoma-, coniothyrium-, microsphaeropsis-, or pyrenochaeta-like (Jaklitsch et al., 2002; Akulov and Hayova, 2016; Hyde et al., 2017; Doilom et al., 2018), which is also similar to coelomycetous asexual morph of *Roussoella* (Hyde et al., 2017). Hyde et al. (2017) tentatively placed *Immotthia* in Roussoellaceae based on the morphological similarity to *Roussoella.* However, *Immotthia* is phylogenetically distinct from *Coniothyrium* (Coniothyriaceae) and *Roussoella* (Roussoellaceae). The genus is transferred from Roussoellaceae to Dictyosporiaceae in this study. The coelomycetous asexual morph of *Immotthia* was found on natural substrates associated with the sexual morph. However, the link between sexual and asexual morph of *Immotthia* has not yet been proven, although Jaklitsch et al. (2002) attempted to elucidate the connection based on cultural experiments. The ascospores of *Immotthia* do not germinate on artificial media due to its obligate parasitic life mode. The connection of the sexual–asexual morph needs to be confirmed based on DNA sequence data obtained from direct DNA extraction of fruiting bodies as well as on culture-based studies.

Asexual morphs of most genera in Dictyosporiaceae are hyphomycetes, except for *Immotthia*, *Pseudocoleophoma*, *Pseudocyclothyriella*, and *Pseudoconiothyrium* which are coelomycetous asexual morphs (Tanaka et al., 2015; Crous et al., 2019; Hongsanan et al., 2020). Based on morphological characteristics, *Immotthia* is most similar to *Pseudoconiothyrium* in having hyaline, smooth, doliiform to ampulliform, phialidic conidiogenous cells and ellipsoidal, brown conidia (Crous et al., 2019). However, the genus can be distinguished from *Pseudoconiothyrium* by multiloculate, smaller conidiomata, smooth-walled conidia, whereas *Pseudoconiothyrium* has uniloculate, larger conidiomata, verrucose conidia (Crous et al., 2019). Two strains of *Pseudocoleophoma clematidis* formed a stable clade basal to *Immotthia* and separated from the main clade of *Pseudocoleophoma* in all analyses (Figure 4 and Supplementary Figures 1–3). We, therefore, re-examined the holotype specimen of *P. clematidis* and found that the species was morphologically different from the other *Pseudocoleophoma* (see Table 3). Thus, *Pseudocyclothyriella* is introduced herein based on the evidence from both morphology and phylogeny. *Pseudocyclothyriella* is also similar to *Pseudoconiothyrium* but differs in having thick-walled, scleroplectenchymatous cells of pycnidial wall. Based on our current phylogenetic results, *Immotthia* and *Pseudocyclothyriella* are clearly distinct from *Pseudoconiothyrium*.

Only four genera in Dictyosporiaceae have been reported for their sexual morphs, viz. *Dictyosporium* Corda, *Gregarithecium* Kaz. Tanaka and K. Hiray., *Immotthia*, and *Pseudocoleophoma*. *Immotthia* is morphologically different from these three genera in having ellipsoidal to fusiform, light brown to reddish brown, asymmetrical ascospores, lacks mucilaginous sheath, and exhibits a hyperparasitic life mode (Jaklitsch et al., 2002; Hyde et al., 2017). On the other hand, the other three genera have hyaline, fusiform to narrowly fusiform ascospores, with or without mucilaginous sheath and have been reported as saprobes in terrestrial and freshwater habitats (Tanaka et al., 2015; Boonmee et al., 2016). The familial descriptions for Dictyosporiaceae have previously been restricted to hyphomycetous asexual morphs (Boonmee et al., 2016; Hongsanan et al., 2020). We recommend that the descriptions and illustrations of Dictyosporiaceae should include both coelomycetous and hyphomycetous asexual morphs.

## Data Availability Statement

The datasets presented in this study can be found in online repositories. The names of the repository/repositories and accession number(s) can be found in the article/Supplementary Material.

## Author Contributions

H-BJ and RP: conceptualization, data curation, and formal analysis. SL, SCK, and RP: funding acquisition. H-BJ, RP, CP, and MD: investigation, methodology, and writing—original draft. RP and NS: project administration. SL, SCK, RJ, and PK: supervision. NS, PK, RJ, SL, and SCK: writing—review and editing. All authors contributed to the article and approved the submitted version.

## Conflict of Interest

The authors declare that the research was conducted in the absence of any commercial or financial relationships that could be construed as a potential conflict of interest.

## Footnotes

1 https://blast.ncbi.nlm.nih.gov/Blast.cgi

2 https://mafft.cbrc.jp/alignment/server/

3 http://tree.bio.ed.ac.uk/software/figtree/

4 https://www.treebase.org/, submission ID: 27606.

## Supplementary Material

Supplementary Figure 1 RAxML tree based on ITS, LSU and TEF1-α sequence matrix represented the phylogenetic relationships of taxa in Dictyosporiaceae. The tree is rooted to *Periconia igniaria* (CBS 845.96 and CBS 379.86). Bootstrap support values for ML are indicated above the nodes. Ex-type strains are in black bold and the new species and new combinations are indicated in blue bold.

Supplementary Figure 2 Phylogram generated by Bayesian inference (BI) analysis based on a combined ITS, LSU, SSU and TEF1-α sequence matrix represented the phylogenetic relationships of taxa in Dictyosporiaceae. The tree is rooted to *Periconia igniaria* (CBS 845.96 and CBS 379.86). Bayesian posterior probabilities are indicated above the nodes. Ex-type strains are in black bold and the new species and new combinations are indicated in blue bold.

Supplementary Figure 3 RAxML tree based on LSU, SSU, TEF1-α, RPB2 and ITS sequence matrix represented the phylogenetic relationships of *Immotthia* in Dictyosporiaceae with other families in Pleosporales. The tree is rooted to *Capondium coffeae* (CBS 147.52). Bootstrap support values for ML are indicated above the nodes. The placement of *Immotthia* in Dictyosporiaceae is indicated by yellow background.
