# Supplementary figures and images for: Reappraisal of Immotthia in Dictyosporiaceae, Pleosporales: Introducing Immotthia bambusae sp. nov. and Pseudocyclothyriella clematidis comb. et gen. nov. Based on Morphology and Phylogeny
Source: Front Microbiol. 2021 May 7;12:656235. doi: 10.3389/fmicb.2021.656235 (PMC8137994; doi:10.3389/fmicb.2021.656235)

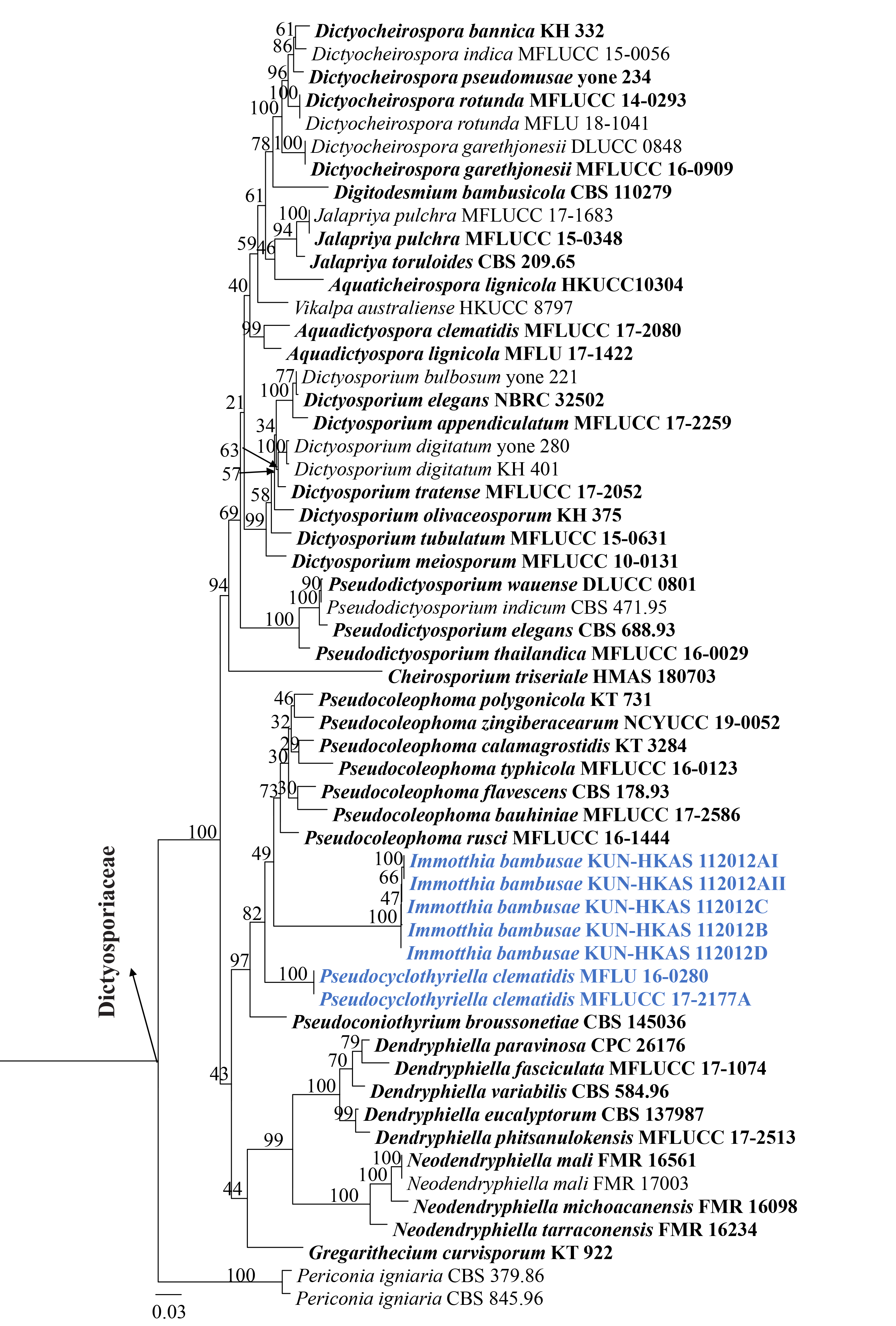

Supplement: Supplementary Figure 1 — RAxML tree based on ITS, LSU and TEF1-α sequence matrix represented the phylogenetic relationships of taxa in Dictyosporiaceae. The tree is rooted to Periconia igniaria (CBS 845.96 and CBS 379.86). Bootstrap support values for ML are indicated above the nodes. Ex-type strains are in black bold and the new species and new combinations are indicated in blue bold. [file Image_1.TIF]

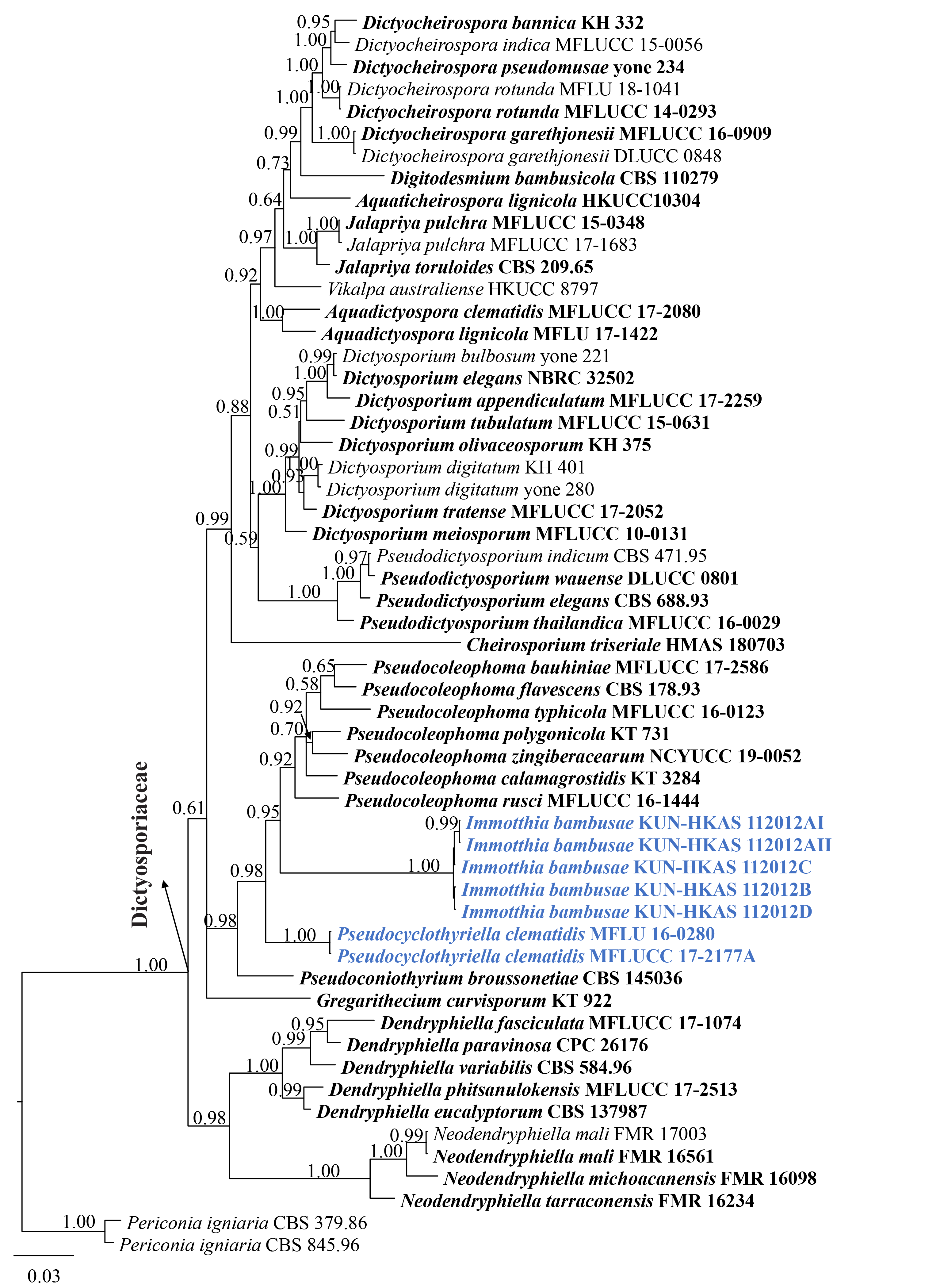

Supplement: Supplementary Figure 2 — Phylogram generated by Bayesian inference (BI) analysis based on a combined ITS, LSU, SSU and TEF1-α sequence matrix represented the phylogenetic relationships of taxa in Dictyosporiaceae. The tree is rooted to Periconia igniaria (CBS 845.96 and CBS 379.86). Bayesian posterior probabilities are indicated above the nodes. Ex-type strains are in black bold and the new species and new combinations are indicated in blue bold. [file Image_2.TIF]

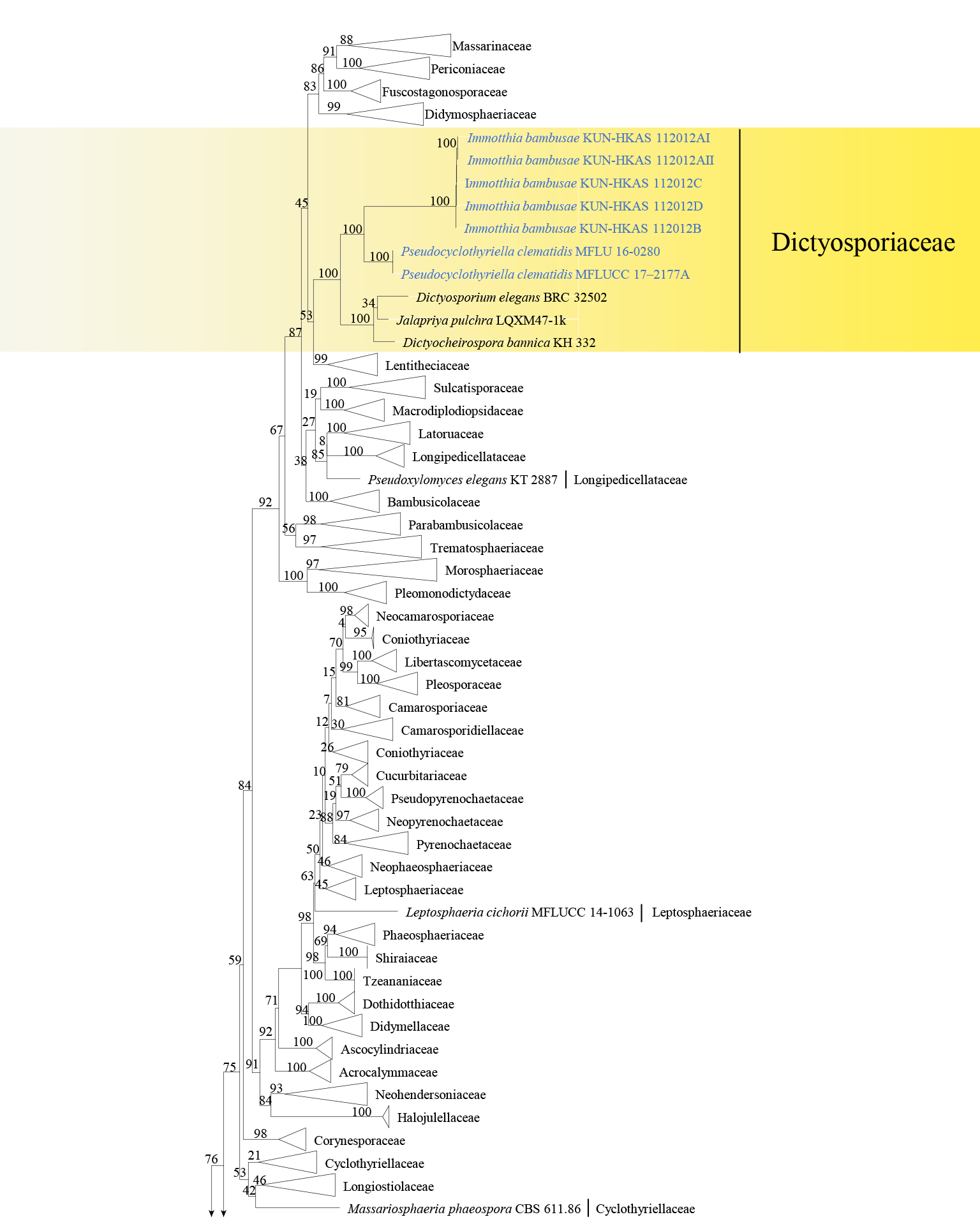

Supplement: Supplementary Figure 3 — RAxML tree based on LSU, SSU, TEF1-α, RPB2 and ITS sequence matrix represented the phylogenetic relationships of Immotthia in Dictyosporiaceae with other families in Pleosporales. The tree is rooted to Capondium coffeae (CBS 147.52). Bootstrap support values for ML are indicated above the nodes. The placement of Immotthia in Dictyosporiaceae is indicated by yellow background. [file Image_3.TIF]

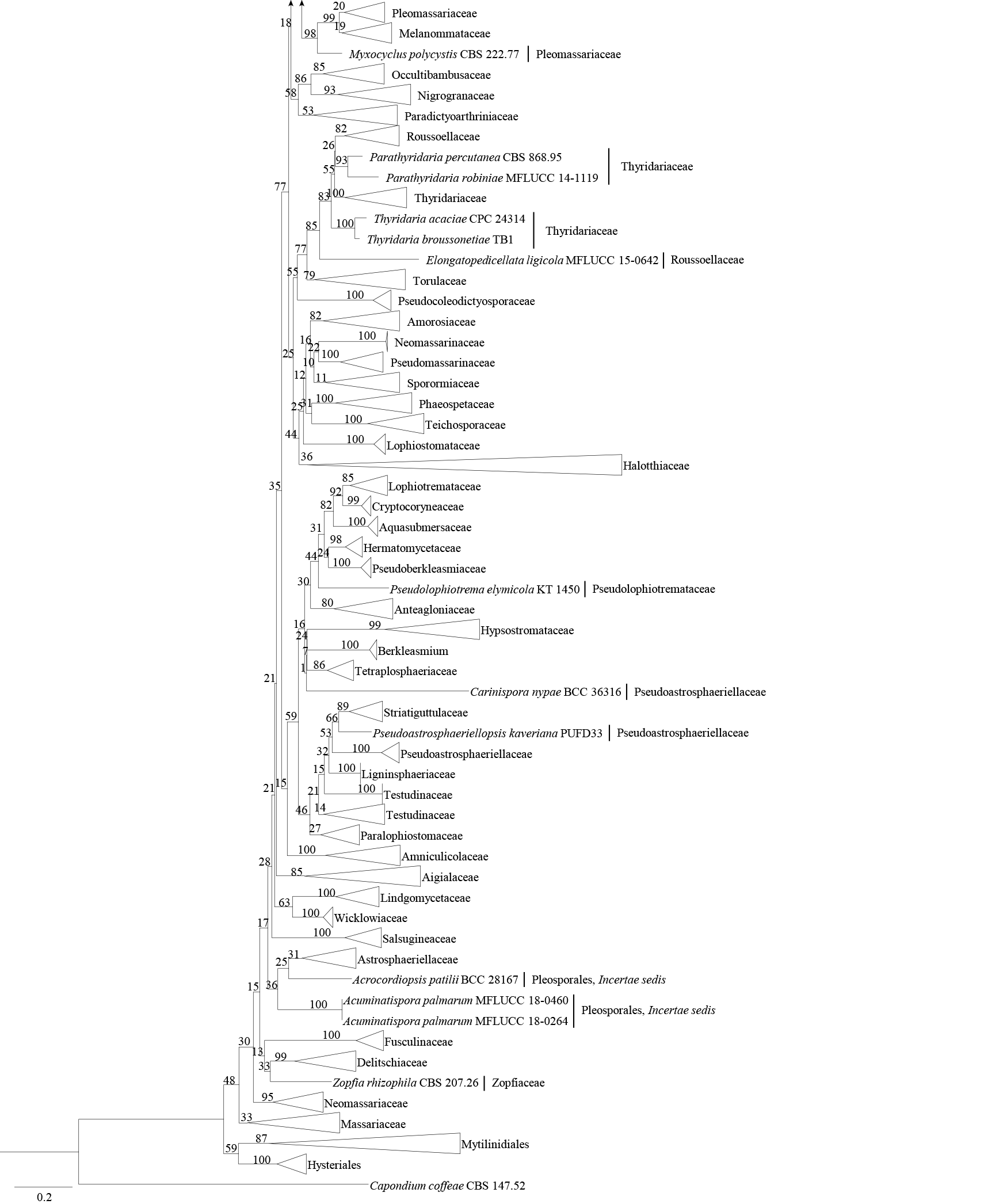

Supplement: Supplementary file 4 [file Image_4.TIF]
